# Progress of Materials and Devices for Neuromorphic Vision Sensors

**DOI:** 10.1007/s40820-022-00945-y

**Published:** 2022-10-15

**Authors:** Sung Woon Cho, Chanho Jo, Yong-Hoon Kim, Sung Kyu Park

**Affiliations:** 1 Department of Electrical and Electronics Engineering, Chung-Ang University, Seoul, 06974 Republic of Korea; 2 Department of Advanced Components and Materials Engineering, Sunchon National University, Sunchŏn, Jeonnam 57922 Republic of Korea; 3 School of Advanced Materials Science and Engineering, Sungkyunkwan University, Suwon, 16419 Republic of Korea; 4 SKKU Advanced Institute of Nanotechnology (SAINT), Sungkyunkwan University, Suwon, 16419 Republic of Korea

**Keywords:** In-sensor computing, Near-sensor computing, Neuromorphic vision sensor, Optoelectronic synaptic circuit, Optoelectronic synapse

## Abstract

The latest developments in bio-inspired neuromorphic vision sensors can be summarized in 3 keywords: smaller, faster, and smarter. (1) Smaller: Devices are becoming more compact by integrating previously separated components such as sensors, memory, and processing units. As a prime example, the transition from traditional sensory vision computing to in-sensor vision computing has shown clear benefits, such as simpler circuitry, lower power consumption, and less data redundancy. (2) Swifter: Owing to the nature of physics, smaller and more integrated devices can detect, process, and react to input more quickly. In addition, the methods for sensing and processing optical information using various materials (such as oxide semiconductors) are evolving. (3) Smarter: Owing to these two main research directions, we can expect advanced applications such as adaptive vision sensors, collision sensors, and nociceptive sensors. This review mainly focuses on the recent progress, working mechanisms, image pre-processing techniques, and advanced features of two types of neuromorphic vision sensors based on near-sensor and in-sensor vision computing methodologies.

## Highlights

- The neuromorphic vision sensors for near-sensor and in-sensor computing of visual information are implemented using optoelectronic synaptic circuits and single-device optoelectronic synapses, respectively.
- This review focuses on the recent progress, working mechanisms, and image pre-processing techniques about two types of neuromorphic vision sensors based on near-sensor and in-sensor vision computing methodologies.

## Introduction

**Fig. 1:**
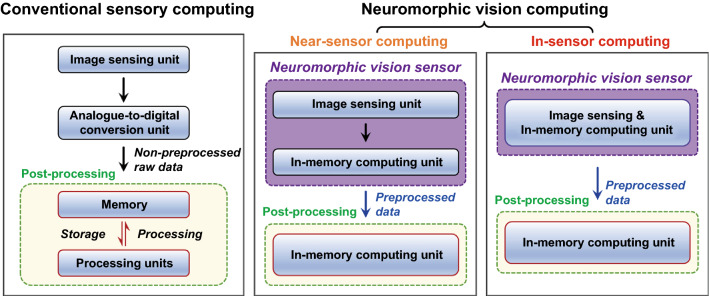
Image processing flow of conventional sensory computing and neuromorphic vision computing

The development of advanced optoelectronic vision sensors for enabling advanced image recognition of visual information and data pre-processing will accelerate advances in machine vision and mobile electronics. In contrast to conventional sensory computing methods including analogue-to-digital signal conversion and digital-logic computing tasks (i.e., von Neumann computing), neuromorphic vision computing can dramatically improve the energy efficiency and data processing speed by minimizing unnecessary raw data transmissions between front-end photosensors and back-end post-processors (Fig. 1) [1–4]. Neuromorphic vision sensors are generally appropriately designed for neuromorphic vision computing tasks, such as denoising, edge enhancement, spectral filtering, and the recognition of visual information. Depending on whether in situ pre-processing is possible, the approaches can be divided into methods using near-sensor and in-sensor computing processors [5]. In a near-sensor computing method, the image sensor for capturing visual information and in-memory computing processor for pre-processing the captured image exist separately. An in-memory computing processor can simultaneously perform memory and computing tasks based on analog memory functions [6]. Neuromorphic vision sensors for in-sensor computing can be built with single-element image sensors which allows the both reception of visual information and execution of in-memory computing processes in the same device. This is an ideal case for future vision-computing systems in artificial intelligence machines and mobile electronic devices.

**Fig. 2:**
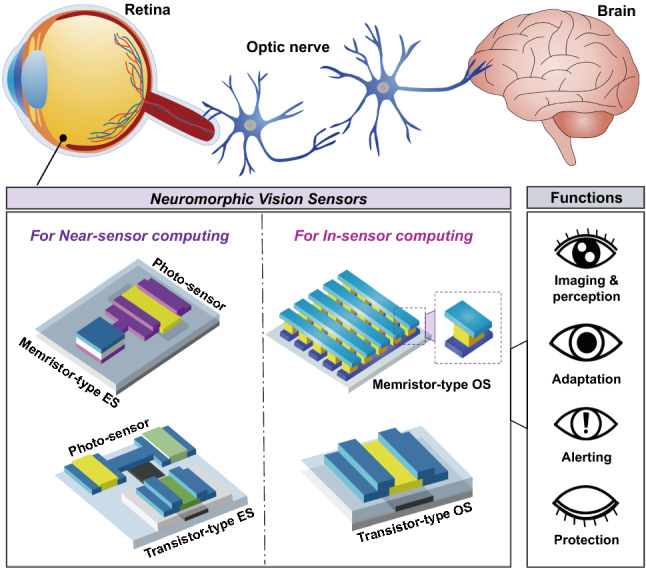
Bio-inspired neuromorphic vision sensors; optoelectronic synaptic circuits (OSCs) operating in near-sensing computing manner and optoelectronic synapses (OSs) operating in in-sensor computing manner

The neuromorphic vision sensors for near-sensor and in-sensor computing can be implemented using (i) optoelectronic synaptic circuits (OSCs) and (ii) single-device optoelectronic synapses (OSs), respectively (Fig. 2) [7, 8]. OSCs are designed for near-sensor vision computing (i.e., near-sensor computing processors), and can be built by interconnecting a series of discrete functional units such as photosensors and electrical synapses (ESs). Artificial ESs are the most important components for performing both memory and computational tasks (i.e., in-memory computing), and for improving the data processing speed and power consumption efficiency during image processing. The artificial synapses for in-memory computing must have conductance switching capabilities and analog storage states. These analog memory functions can be evaluated according to the short-term plasticity, long-term potentiation, long-term depression, paired pulse facilitation, paired pulse depression, spike time-dependent plasticity, spike number-dependent plasticity, and spike frequency-dependent plasticity [9, 10]. The artificial ESs use positive and negative bias spikes as electrical programming means for exhibitory and inhibitory conductance updates. ESs are usually produced using two-terminal memristors and three-terminal transistor device structures. Memristors generally benefit from high-density arrays, easy fabrication processes, and high programming speeds. In contrast, transistor-type ESs are emerging as a counterpart option, with advantages such as energy-saving computing, uniform device performance, nondestructive readouts, linear/symmetric conductance switching, and high cyclic stability. Next, OSs were initially designed as optoelectronic in-memory computing hardware for modulating conduction states by using optical signals as a programming means. However, OSs have recently attracted attention as neuromorphic vision sensors for in-sensor computing, as they can perform both optical sensing and in-memory computing of visual information in one device (i.e., an in-sensor computing processor). They can be fabricated using device structures of two-terminal memristors and three-terminal transistors. These OSs have unique functional sites for optical responses and conductance switching, depending on the device architecture. For memristor-type OSs, the photoresponsive space is located between the transparent conductive top electrode (TE) and metallic bottom electrode (BE). In the case of a transistor-type OS, the photoresponsive layer is mainly located in the channel region between the source and drain electrodes, where there is an extra gate electrode to engineer the conductance switching performance. Details of the various materials and device structures of neuromorphic vision sensors, including the OSC and OS, will be reviewed below.

In this review, we focus on the recent advances in neuromorphic vision sensors for the machine vision computing systems essential to autonomous vehicles, intelligent robots, and mobile electronics. Section 2 provides an overview of the material design, device architecture, and operating mechanism of an OSC designed as a near-sensor computing processor for visual information. Section 3 details the material design, device architecture, and operating mechanism of a single-element OS designed as an in-sensor computing processor. Section 4 reviews the challenging evolution of neuromorphic vision sensors beyond conventional capabilities and imaging. Finally, the current status and problems of neuromorphic vision sensors are briefly summarized, and their prospects are discussed.

## Neuromorphic Vision Sensor for Near-Sensor Vision Computing

The OSCs designed for near-sensor vision computing (i.e., near-sensor computing processors) can be built by interconnecting a series of discrete functional units, such as photosensors and ESs. The photosensors and photovoltaic transducers used in traditional visual sensory computing are interchangeable in OSCs. This can greatly reduce the burden of developing neuromorphic vision sensors for the imaging, recognition, and pre-processing of image information. In a conventional sensory system, a logic-based computing unit and digital memory unit are required for processing the acquired image information. However, in the case of an OSC, a single artificial ES with an in-memory computing function can replace them, thereby improving the data processing speed and power consumption efficiency when processing visual information. This section reviews the pioneering OSCs and neuromorphic image-recognition results regarding near-sensor vision computing. Then, we take a closer look at the hardware configuration and operating mechanism of the ES, a key component of the near-field sensor computing processor.

### Neuromorphic Image Perception Using Optoelectronic Synaptic Circuit

OSCs can demonstrate neuromorphic image recognition through a series of near-sensor computing processes, including image sensing in photosensors, photobias conversion in photovoltaic transducers, and bias-driven in-memory computing in artificial ESs [11–14]. When the optical information from the incident image arrives in the individual OSC of each pixel of the neuromorphic vision sensor, each photosensor experiences a transient change in the channel conductance while receiving the optical input. The change in the light-driven conductance of the photosensors is converted into the form of a bias signal in the photovoltaic transducer. Then, the ES experiences historical analog conductance switching depending on the frequency and intensity of the translated bias spike. Finally, by mapping the switched conductance values of the ESs located in each pixel, well-refined images can be acquired through neuromorphic vision perception with improved computing speed and power-consumption conditions.

**Fig. 3:**
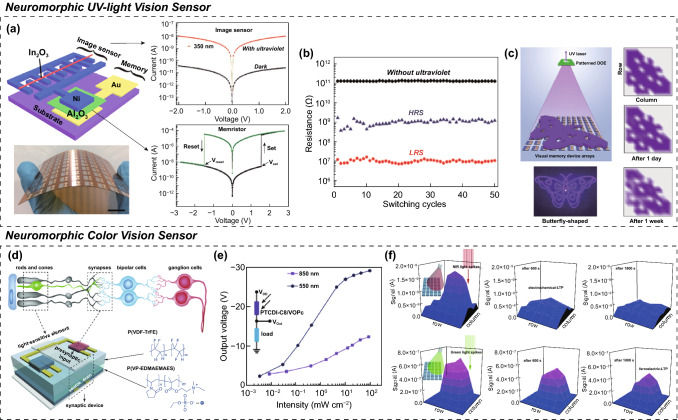
OSC for near-sensor computing system; circuit configuration and neuromorphic vision perception. Neuromorphic ultraviolet (UV)-light vison sensor; **a** circuit schematic consisting of photo-sensor and memristor-type electrical synapse (ES), **b** conductance switching behavior in electrical memristor and its operation mechanism, and **c** neuromorphic perception of UV-light butterfly pattern image. Neuromorphic color-vison sensor; **d** circuit schematic consisting of photo-sensor, load, and transistor-type ES, **e** photo-to-bias conversion performance of photovoltaic divider, and **f** neuromorphic perception of color and near-infrared (IR) images. **a**–**c** Reproduced with permission from Ref. [11]. Copyright @ 2018, Wiley-VCH. **d**–**f** Reproduced with permission from Ref. [14]. Copyright @ 2018, Wiley-VCH

In a pioneering work, Shen et al. [11] reported an OSC capable of the neuromorphic perception processing of ultraviolet (UV) light images. It was constructed by directly connecting a diode-type photosensor using an In_2_O_3_ thin film with a wide bandgap and a conductive filament (CF) memristor-type ES using Ni/Al_2_O_3_/Au layers (Fig. 3a). When the OSC was exposed to UV light, the partial voltage assigned to the photosensor unit decreased rapidly with light-induced transient changes in the conductance of the In_2_O_3_. Conversely, a sufficient residual voltage above the threshold was provided to the memristor device and induced analog switching and nonvolatile updating of the memristor conductivity with the Ni CF formation and growth (Fig. 3b). By mapping the conductance values of the memristors at each pixel, well-refined visual data of the incident UV light images were obtained using a neuromorphic vision sensor array (Fig. 3c). However, this approach cannot detect color image information because the In_2_O_3_ semiconductor has a wider bandgap than the visible light energy. Therefore, it is disadvantageous to various image recognition.

To overcome this issue, several investigations have been conducted to develop OSCs for the near-sensor computing of color image data [13, 14]. Color-perceive neuromorphic vision sensors can be realized by interconnecting color-responsive photosensor with color-inactive ES. Liu et al. [14] reported a color-perceiving OSC fabricated using a color-responsive photovoltaic divider and transistor-type ES (Fig. 3d). Instead of directly connecting the photosensor and artificial synapse, they designed an advanced OSC similar to an inverter circuit configuration and arranged the photosensor, load resistor, and artificial synapse in the proper positions. When the photovoltaic divider was exposed to color images, the color-responsive photosensor experienced a temporary conductance change. In contrast, since the load resistor uses a color-inactive semiconducting channel, the original conductivity of the load resistor can be maintained regardless of whether a color light irradiation. Depending on the ratio of the electrical conductivity between the photosensor and load resistor, an appropriate output voltage was transferred from the photovoltaic divider to the region of the output voltage electrode connected to the gate terminal of the transistor-type ES (Fig. 3e). The converted voltage signal then induced conductance switching for the artificial synapse, as electrical programming input. Here, it was confirmed that the amplitude of the output voltage converted in the photovoltaic divider and the conductance value of the transistor-type ES increased when the light signals having a shorter wavelength or a higher intensity were incident. However, because only one photosensor is contained, both short wavelength but low intensity light and long wavelength but high intensity light caused small output voltage of similar range on the voltage divider. Therefore, it is a little difficult to recognize the difference between the above-mentioned two images through this OSC-type neuromorphic vision sensor. However, for high-intensity incident light condition, more energetic green image with shorter wavelength (*λ* = 550 nm) than NIR image (*λ* = 850 nm) could cause much higher output voltage in the photovoltaic divider, which generated higher conductance change of longer duration for electrical ESs. Finally, green color image with high light intensity could be successfully perceived and memorized in the color-responsive OSC designed for near-sensor computing (Fig. 3f). However, there are several intrinsic problems, such as many parts, complex manufacturing processes, and low device density. For high-resolution and on-chip neuromorphic vision sensing, vertical-integrated approaches are more advantageous than planar-integrated approaches, owing to the feasibility of fabricating neuromorphic vision sensors with a smaller chip size. However, this requires advanced processing technologies and device architectures.

### Neuromorphic In-Memory Computing Using Electrical Synapse

Artificial synapses have been designed as hardware processors for in-memory computing, e.g., for simultaneously storing and computing incoming data. Artificial synapses with a biological information computing methodology (rather than conventional digital logic-based computing) can dramatically improve the computing speed and power consumption efficiency of information processing for vast amounts of unstructured data such as images, videos, sound, and languages. Many artificial synapses have been steadily developed based on memristor- and transistor-based device structures. As mentioned in the previous section, in OSCs, artificial ES devices represent the most important and essential parts of the imaging and pre-processing of the image information. This section reviews the device configurations and operating mechanisms of ESs.

#### Transistor-Type Electrical Synapses

**Fig. 4:**
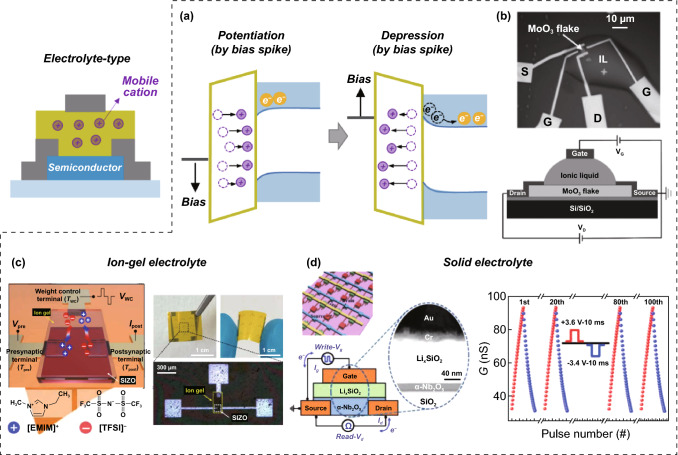
Electrolyte transistor-type ES; **a** operation mechanism, **b** ionic liquid electrolyte-based transistor-type ES, **c** ion-gel electrolyte-based transistor-type ES, and **d** inorganic solid electrolyte-based transistor-type ES. **b** Reproduced with permission [19]. Copyright @ 2017, Wiley-VCH. **c** Reproduced with permission from Ref. [20]. Copyright @ 2021, AAAS. **d** Reproduced with permission from Ref. [27]. Copyright @ 2020, Wiley-VCH

All transistor-type ESs have traditional transistors including insulating gate dielectrics, semiconducting channels, and conductive three-terminal electrodes (Fig. 4). However, each transistor-type ES has unique materials and/or a unique device design to perform conductance switching and analog conductance updating functions using electrical bias programming methods [9, 15]. Based on the operation mechanisms of transistor-type ESs, they can be classified into electrolyte, floating gate (FG), and ferroelectric-type ESs.

##### Electrolyte Transistor-type ES

Electrolyte transistor-type ESs can be manufactured by introducing electrolytes with high capacitance into the dielectric region. Those electrolytes are synthesized by incorporating mobile cations into an insulating matrix via solution processes. Generally, mobile cations with small radii are used, such as protons (H^+^), alkali metals (Li^+^, Na^+^, and K^+^), and alkaline earth metal ions (Ca^2+^ and Mg^2+^). According to the matrix phase, electrolytes can be classified into liquid (ionic liquid), gel (ionic gel), and solid electrolytes (polymeric electrolyte and inorganic electrolyte) [16, 17]. When a positive bias input is applied to the gate terminal, the mobile cations in the electrolyte start to drift to the electrolyte/channel interface, owing to the positive bias-induced electric field (Fig. 4a). The mobile cations accumulate near the electrolyte/channel interface, resulting in a nonuniform concentration distribution of the mobile cations in the electrolyte (and creating a concentration gradient). In the case of n-type channel transistors, these sequentially accumulated metal cations produce excitatory changes (i.e., potentiation) in the channel conductance with the formation of electron double layers and electrochemical doping reactions [18]. Even when the bias is no longer applied, the updated conduction state is maintained for a long time, as the diffusion process requires a significant amount of time for the concentration gradient of the mobile cations to dissipate. However, the mobile cations accumulated at the electrolyte/channel interface gradually return to their original uniform state when an opposite bias input is applied. Therefore, inhibitory conductance switching (i.e., depression) can be achieved can be achieved by applying a programming bias spike of opposite polarity to the gate terminal.

According to many surveys, electrolyte transistor-type ESs have been manufactured using a fluid ion liquid and sticky ion-gel-type electrolyte. Aqueous solvents and gel polymers are mainly utilized as matrices for ion-liquid and ion-gel electrolytes. Sun et al. reported an ionic liquid transistor-type ES using 1-Ethyl-3-methylimidazolium bis(trifluoromethylsulfonyl)imide (EMIM-TFSI) and an aqueous solvent. The conductivity of n-type MoO_3_ semiconductors can be modulated to a more conductive (resistive) state through excitatory (inhibitory) updates, owing to the electrochemical doping reaction between the MoO_3_ and protons (Fig. 4b) [19]. Park et al. [20] reported an ion-gel transistor-type ES using an ion-gel electrolyte composed of EMIM-TFSI and a gel matrix (Fig. 4c). The mechanical flexibility of the gel allowed for the reliable fabrication and operation of ion-gel-based ESs on plastic substrates [20–23]. With this flexible in-memory computing device, tactile information from the human hand could be preprocessed directly, without the aid of an external computer device. However, liquid and gel electrolytes make it difficult to manufacture high-density device arrays owing to their fluid and sticky mechanical properties, and their easy chemical modification makes it difficult to secure operational stability. To address these challenges, robust solid electrolytes containing insulating solid matrices such as polyethylene oxide (PEO), silica (SiO_2_), and alumina (Al_2_O_3_) have been actively explored. In practice, various electrolyte transistor-type ESs have been developed using a solid electrolyte with a combination of a solid matrix (polymeric and inorganic matrix) and mobile cations: PEO + Li, SiO_2_ + H, SiO_2_ + Li, etc., [24–27]. For example, Liu et al. [27] reported an all-solid-state transistor-type ES using an inorganic solid electrolyte comprising inorganic SiO_2_ and Li^+^ cations (Fig. 4d). Depending on the programming cycle of bias input, the channel conductivity of the n-type Nb_2_O_5_ semiconductor could be gradually modulated and exhibited a good linear update trend, owing to the use of the heavy Li^+^ cations (as opposed to) light protons.

The computing performance of electrical synapses that serve as in-memory computing hardware in OSC-type near sensors can be evaluated based on the following factors: (i) programming speed, (ii) programming power, (iii) nonvolatile storage capability, (iv) programmable number of conductance, and (v) linearity during conductance update. In the cases of electrolyte transistor-type ESs, their performance will be affected by the matrix phase and mobile cationic species of the electrolyte layer. In terms of programming speed and programming power, using an electrolyte composed of a liquid matrix and small mobile cations is advantageous over using an electrolyte composed of a rigid solid matrix and relatively large cations. This is because small mobile cations can be easily drift from bulk region of the soft electrolyte (i.e., ionic liquid and ion gel) to the channel region when a programming bias is provided to enhance (i.e., potentiation) and decrease (i.e., depression) the channel conductance. Indeed, a longer programming time (i.e., slow programming speed) or higher programming bias conditions (i.e., high power consumption) are required to update the channel conductance when using a solid electrolyte. In contrast, solid electrolyte is advantageous for nonvolatile storage of programmed conductance states. This is because the mobile cations accumulated on one side of the electrolyte by previous bias programming require a lot of time to return to their original position through the solid electrolyte. In fact, compared with the cases using the ionic liquid and ion-gel electrolytes, the electrical synapse using the solid electrolyte showed stable retention of channel conductance updated by bias programming [24–27]. In addition, for circuit-type neuromorphic vision sensors, solid electrolyte-based in-memory devices are expected to be preferred because photosensors and other components must be closely connected for high-resolution image recognition among various electrolytes.

##### FG Transistor-Type ES

**Fig. 5:**
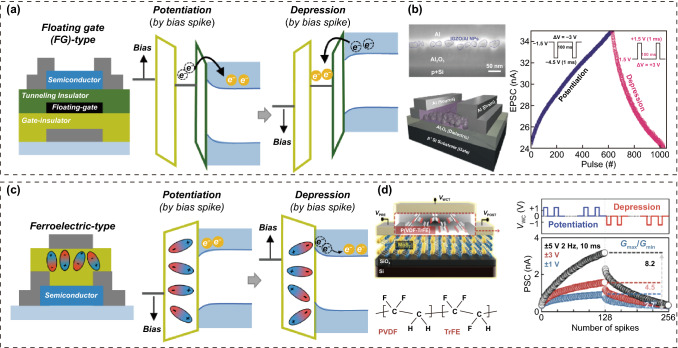
Floating-gate (FG) transistor-type ES; **a** operation mechanism, and **b** Al nanoparticles (NPs)-FG transistor-type ES. Ferroelectric transistor-type ES; **c** operation mechanism and **d** poly(vinylidene fluoride)-trifluoroethylene (PVDF-TrFE) ferroelectric transistor-type ES. **b** Reproduced with permission from Ref. [30]. Copyright @ 2020, Wiley-VCH. **d** Reproduced with permission from Ref. [35]. Copyright @ 2021, Royal society of chemistry publishing

To date, the FG structure is the most widely used architecture for transistor-based synapses, owing to its stable conductance switching and long-term retention characteristics [28, 29]. An FG transistor-type ES can be fabricated by vertically stacking thick blocking dielectrics, metallic FGs, ultrathin tunneling dielectrics, and semiconductor channels (Fig. 5a). Here, the metallic FG region serves as a key part of the channel conductance switching. As in the other cases, electrical bias spikes of different polarities are utilized as the programming sources for the excitatory and inhibitory conductance switching for in-memory computing tasks. When a negative (positive) bias is applied at the gate terminal, electrons (holes) are injected from the FG to the channel region via the Fowler–Nordheim tunneling process. Therefore, the channel conductance can be modulated to a more conductive state by undergoing an excitatory update with an increase in majority carriers. In contrast, when a positive (negative) bias programming input is applied, the majority carriers in the n-type (p-type) channel region move to the FG region. Therefore, the semiconducting channel exhibits a more resistive electrical performance with the reduction of the majority carriers, achieving inhibitory programming.

For long-term charge storage (i.e., nonvolatile storage), it is necessary to use metal material in a low-dimensional form rather than a film form because the charge accumulated in the FG should be less lost through the internal leakage path. Noble metals (e.g., Au, Pt, and Cu) are preferred as FG materials due to their low chemical deformation during device fabrication and operation. Furthermore, ultra-thin tunneling layers with wide band gap insulators (e.g., SiO_2_, Al_2_O_3_, and TiO_2_) between the FG and channel layers are required to maintain the modulated conductance for a long period of time. However, it is difficult to create a uniform ultrathin tunneling layer on the surfaces of metallic nanomaterials. To address these issues, some studies have proposed the intentional use of materials able to easily generate ultrathin oxide layers on their surfaces. Kim et al. [30] reported an FG transistor-type ES using Al–Al_2_O_3_ core–shell nanoparticles (NPs) in the FG region. It was possible to spontaneously form a uniform ultrathin Al_2_O_3_ tunneling layer between the Al NPs and channel layer, owing to the facile oxidation properties. Owing to the electronic band alignment between the Al NP FG and InGaZnO (IGZO) and the n-type semiconductor nature of IGZO, the device could be modulated into more conductive and resistive states by negative and positive bias programming inputs (Fig. 5b). Similarly, Jo et al. [31] reported an FG transistor-type ES manufactured with Ti_3_C_2_T_*x*_ MXene-TiO_2_ core–shell nanosheets in the FG region using a solution coating process. An ultrathin TiO_2_ tunneling layer on the surface of metallic Ti_3_C_2_T_*x*_ MXene could be uniformly generated through a simple oxidation reaction in the atmosphere. In contrast to the Al NP FG/n-type IGZO synaptic transistor, the device could be programmed to conductive and resistive states by positive and negative bias spikes. This was owing to the electronic band alignment between the MXene FG and pentacene, and the p-type semiconductor properties of the pentacene. Generally, FG devices are advantageous for wide conductance switching ranges and nonvolatile long-term storage. When operating as an in-memory computing device, an FG device can exhibit a wide conductance dynamic range while strongly retaining its programmed conductance state. However, for the linear/symmetric conductance update and energy-saving image processing, problems such as abrupt conductance changes and relatively high operating voltage conditions arising from the FG device-based operation mechanism should be improved.

##### Ferroelectric Transistor-Type ES

A ferroelectric transistor-type ES can be fabricated by introducing ferroelectric materials into the gate-dielectric region [32]. Ferroelectric materials with spontaneous electrical polarization capability and multiple domains can provide nonvolatile analog memory functions. When an electrical bias spike is applied to the gate terminal, a portion of the randomly oriented ferroelectric region begins to align in the direction of the external electric field (Fig. 5c). The channel conductance can be gradually updated by changing the polarization of the ferroelectric film. When a positive (negative) gate bias spike is applied, the n-type semiconducting channel shows a gradual increase (decrease) in the channel conductance (Fig. 5d). The updated channel conductance can be maintained owing to the spontaneous electrical polarization of the ferroelectric material. In contrast, an p-type semiconducting channel exhibits a gradual inhibitory (exhibitory) modulation behavior with positive (negative) electrical programming signals.

Polymeric and inorganic ferroelectric materials such as Pb(Zr,Ti)O_3_ (PZT), zirconium-doped hafnia (HZO), and poly(vinylidene fluoride) (PVDF) are widely used in the fabrication of ferroelectric transistor-type ESs [33–35]. Lee et al. [34] reported an oxide-ferroelectric transistor-type ES using HZO (ferroelectric region) and n-type IGZO (channel region). The HZO/IGZO ferroelectric transistor exhibited a counterclockwise hysteresis loop owing to the ferroelectric polarization switching of the HZO. The channel conductance of IGZO can also be modulated to potentiation (depression) states with positive (negative) bias inputs, owing to the spontaneous polarization of the HZO ferroelectric. Inorganic material-based ferroelectric transistors can successfully perform essential channel conductance switching functions for in-memory computing. However, problems remain, such as high power consumption, high-cost vacuum processes, and the rigid mechanical properties of inorganic materials. To address these problems, Park et al. [35] reported polymer-based ferroelectric transistor-type ES using a PVDF-trifluoroethylene P(VDF-TrFE) ferroelectric copolymer. Compared with conventional inorganic ferroelectrics, the P(VDF-TrFE) ferroelectric copolymers are advantageous for the solution coating and low-temperature processes, making them suitable for flexible electronic products. Their ferroelectric performance can be optimized by phase transformations via thermal annealing. In the study, a P(VDF-TrFE) film with excellent crystallinity and *β*-phase domains exhibited the best spontaneous polarization, as the molecular dipoles were aligned perpendicular to the long axis of the polymer chain. The channel conductance of n-type MoS_2_ can be switched to more conductive or resistive states (potentiation and depression states) via the polarization modulation of P(VDF-TrFE) by positive and negative bias inputs, respectively.

Comparing the computing performance of ESs, in terms of nonvolatile storage of the programmed conductance state, the electrolyte transistor-type ESs show unstable properties in programmed conductivity state. This is because the mobile cations accumulated at the channel/electrolyte interface by the drift process during bias programming prefer to return to their original position after programming through the spontaneous diffusion process. In fact, most ionic liquid-based transistor-type ESs exhibit almost volatile storage characteristics owing to the use of liquid matrix. In contrast, FG and ferroelectric transistor-type ESs mainly exhibit much longer nonvolatile retention performances of the programmed conductance state. Transfer of charge trapped in the floating gate through the tunneling barrier to the channel region is an involuntary process that requires external forced action. Therefore, the programmed conductance state can be maintained for a long time if there are no additional high voltage programming attempts. Likewise, reorienting the direction of polarized domains in ferroelectric materials is an involuntary process requiring external bias programming signal. FG and ferroelectric-type ES are disadvantageous for low power and high speed operation compared to electrolyte transistor-type ES. This is because conductance switching methods, such as tunneling of charge carriers and reorientation of ferroelectric domains, require higher voltage conditions and programming time compared to that using drift of mobile cations in the electrolyte.

#### Memristor-Type Electrical Synapses

All memristor-type ESs have a simple two-terminal device configuration consisting of individual electrodes in the upper and lower regions, and an active layer in the middle region. However, each type of memristor-type ES has unique operational strategies for having conductance switching and analog conductance update functions. According to the operation mechanism, they can be classified into electrochemical metallization (ECM), valence change (VC), and phase change (PC) memristor-type ESs [36, 37].

##### ECM Memristor-Type ES

**Fig. 6:**
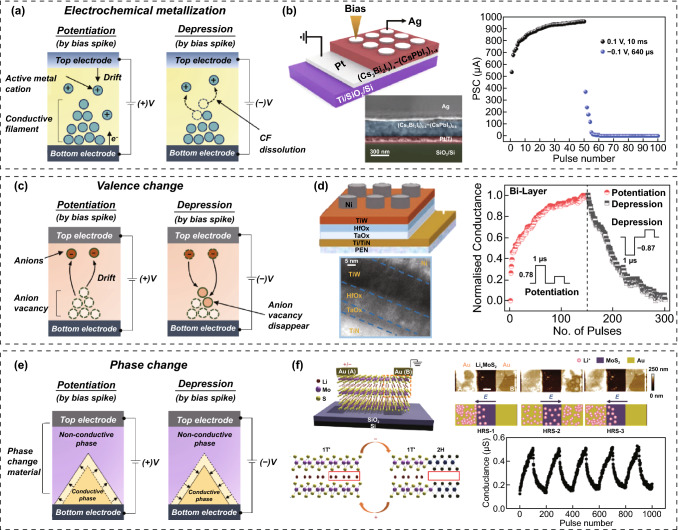
Memristor-type ESs; device configuration, operation mechanism, and electrical conductance switching performance. **a**, **b** electrochemical metallization (ECM) memristor-type ES: **a** operation mechanism and **b** perovskite-based ECM memristor-type ES. **c**, **d** valence change (VC) memristor-type ES: **c** operation mechanism and **d** oxide-based VC memristor-type ES. **e**, **f** phase change (PC) memristor-type ES: **e** operation mechanism and **f** Transition-metal dichalcogenide (TMDC)-based memristor-type ES. **b** Reproduced with permission from Ref. [41]. Copyright @ 2019, Wiley-VCH. **d** Reproduced with permission from Ref. [46]. Copyright @ 2022, Wiley-VCH. **f** Reproduced with permission from Ref. [50]. Copyright @ 2019, Springer Nature

ECM memristor-type ESs can be fabricated by introducing active and inert metals on each side electrode and insulating or semi-insulating dielectrics in the active layer (Fig. 6a). For ECM memristors, the growth and rupture of CFs through electrochemical redox reactions are the main causes of conductance switching behavior. Generally, various insulating and semi-insulating materials (e.g., SiO_2_ Ta_2_O_5_, Ag_2_S, and Cu_2_S) can be used in the active layer [38–40]. Electrochemically active metals (e.g., Ag or Cu) and inactive metals (e.g., Pt and Au) are mainly introduced into the TE and BE, respectively. For example, if there is Ag in the TE and an inert metal in the BE. When a sufficiently positive bias is applied to the TE, Ag is ionized into Ag^+^ by the oxidation reaction triggered at the interface between the TE and the active layer. The Ag^+^ ions move toward the BE, owing to the bias-induced electric field. Subsequently, Ag starts to accumulate at the interface between the active layer and the BE by causing a reduction reaction with the electrons supplied from the BE. Owing to the continuous accumulation of Ag atoms, Ag-based CFs begin to form near the interface, and gradually grow toward the opposite electrode. The growth of the CF induced by the positive electrical bias spike progressively increases the conductivity of the memristor-type ES. In contrast, the conductance can be switched to a lower state under a negative bias spike owing to the rupture of the CF. For example, Jang et al. [41] reported an ECM memristor-type ES using an inorganic perovskite active layer. In this device structure, a (Cs_3_Bi_2_I_9_)_0.4_–(CsPbI_3_)_0.6_ perovskite active layer and polymethyl methacrylate (PMMA) insulating layer were laminated between a Pt BE and Ag TE (Fig. 6b). This memristor-type ES showed essential synaptic behaviors through the ECM mechanism of Ag cations-based CFs. Electrical potentiation and depression operations were demonstrated by applying positive and negative programming pulses.

##### VC Memristor-Type ES

For the fabrication of VC memristors, inert metals (e.g., Pt, TiN, and Au) or conductive oxides [e.g., InSnO (ITO) and InZnO (IZO)] have been employed at the TE and BE electrodes (Fig. 6c). Various metal oxides (e.g., TiO_*x*_, TaO_*x*_, WO_*x*_, SrTiO_3_, and Pr_0.7_Ca_0.3_MnO_3_) [41, 42] and perovskites (e.g., MAPbBr_3_, CsPbBr_3_, and Cs_3_Cu_2_I_5_) [43, 44], including weakly bonded anions, can be utilized in the active layer of VC memristor-type ESs. When a bias is applied to a material in which cations and anions are weakly coupled, highly charged anions (X^M−^) and anion voids (*V*_*X*_) can be generated inside the active layer [45]. When a positive bias is applied to the TE, a large number of bias-induced X^M−^ ions (e.g., O_2_^−^, Br^−^, and I^−^) and *V*_*X*_ (e.g., *V*_O_, *V*_Br_, and *V*_*I*_) appear in the active layer. Subsequently, the X^M−^ ions migrate to the TE, creating an anion-rich compositional region at the interface of the active layer and TE. Therefore, the metal cations located in the anion-rich compositional region experience a change in the valence state, leading to a new compositional interfacial layer. Furthermore, the *V*_*X*_ migrates to the opposite electrode, leading to the growth of conical *V*_*X*_ CFs and a higher conductive conduction state. In contrast, when a negative bias spike is applied to the BE, the X^M−^ and *V*_*X*_ gradually return to the active layer, leading to inhibitory conductance switching. In other words, for VC memristors, the formation and rupture of interfacial layers with locally different compositions according to VCs of the metal cations in the active layer are the main causes of the conductivity switching behaviors. For example, Tseng et al. [46] reported a VC memristor-type ES using HfO_*x*_/TaO_*x*_ bilayers (Fig. 6d). It showed synaptic potentiation and depression behaviors from stimulation with electrical positive and negative bias pulses, respectively. Here, the excitatory response was attributed to the generation of *V*_*O*_-based CFs in the metal oxide bilayer, whereas the inhibitory response was owing to the cleavage of *V*_*O*_-based CFs.

Generally, ECM and VC devices have high scalability, ultra-low operating voltages, and wide conductance switching ranges owing to similar CF-based operational mechanisms. Therefore, it is expected that ECM and VC ESs will provide advantages such as high pixel density, low power consumption, and a wide sensing dynamic range in OSCs for neuromorphic image processing. However, in the future, the serious problems arising from CF-based operating mechanisms must be addressed, including device-to-device non-uniformity, nonlinear and asymmetric conductance update characteristics, and performance differences during repeated cycle operations [47].

##### PC Memristor-Type ES

The reversible resistive-to-conductive phase transition of PC materials is a major switching principle for PC memristor-type ESs (Fig. 6e). They can be fabricated by introducing phase-change materials (e.g., Ge_2_Sb_2_Te_5_ (GST) and Li-incorporated MoS_2_) between two metallic electrodes [48–50]. Their conductance can be modulated by controlling portions of the conductive and resistive phases using an electrical bias input. For GST, the individual crystalline and amorphous phases represent conductive and insulating electrical characteristics, respectively. A crystallization of the amorphous region can occur when the local heat generated by the electrical bias exceeds the crystallization temperature. In contrast, when the temperature exceeds the melting point, the crystalline region melts and is quenched into the amorphous phase. Therefore, the conductivity of PC memristors can be modulated by engineering localized thermal heating under appropriate electrical bias conditions for excitatory and inhibitory conductivity updates. For Li-incorporated MoS_2_, the individual 1T′ and 2H phases of MoS_2_ exhibit conductive and semiconducting electrical properties, respectively (Fig. 6f) [50]. In particular, the 1T′ phase can be formed by electrochemically intercalating Li into the 2H phase by applying an electrical bias. Therefore, the ratio of the 1T′ and 2H phases can be modulated by reversibly inserting and extracting Li using an electrical programming bias. Accordingly, the conductance of the MoS_2_ device can be gradually increased and reduced by applying electrical positive and negative bias pulses, respectively, resulting in potentiation and depression behaviors.

## Neuromorphic Vision Sensor for In-Sensor Vision Computing

Originally, OSs were developed as optoelectronic in-memory computing devices for using optical signals as programming means, with the advantages of high-speed signal transmission and wide bandwidth. However, recently, the OS has been attracting increased attention as a neuromorphic vision computing processor for the in-sensor computing of visual data. It can act as an all-in-one neuromorphic vision sensor for simultaneously performing image reception and memory computing tasks on the same device. Several innovative OS-type neuromorphic vision sensors have been developed. This section reviews pioneering OSs and the perception performance of neuromorphic vision sensors for in-sensor vision computing. It then examines the hardware configurations and operating mechanisms of OSs in detail.

### Neuromorphic Image Perception using Optoelectronic Synapse

An all-in-one neuromorphic vision sensor should be able to simultaneously perform image sensing and neuromorphic in-memory computing during the vision computing of image data. Therefore, it must include functional areas for photosensing, conductance switching, and analog update functions [51–56]. When visual information arrives in the OS array, each OS with a light-absorbing site experiences a change in the channel conductance. In contrast to conventional photosensors, which exhibit transient conductance changes to light inputs, OSs can maintain altered conductance states after the light input has ended. Several resources are available for maintaining the changed conductance. Furthermore, the altered conductance state can be additionally updated via following image exposures, owing to the existence of the analog conductance states. By mapping the updated conductance values of the OS array, an incident image can be captured and converted into a more refined image.

**Fig. 7:**
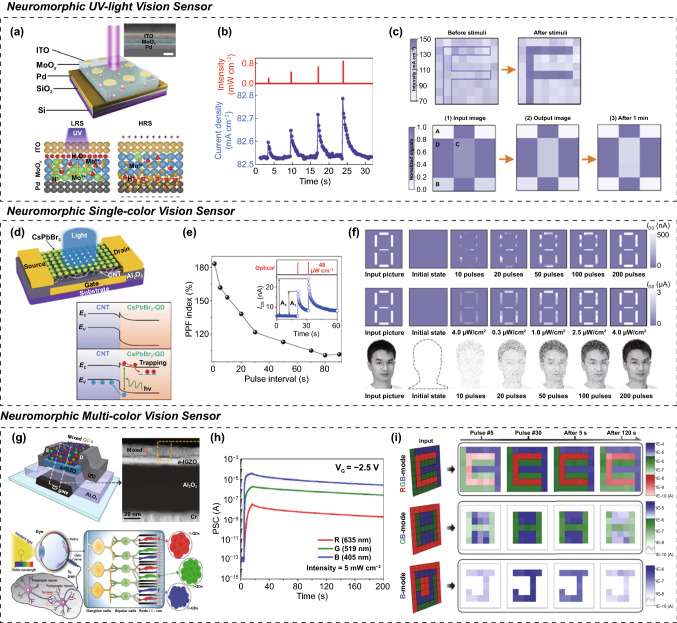
OSs for in-sensor computing system; device configuration; and neuromorphic vision perception. **a**–**c** Neuromorphic UV-light vison sensor; **a** device schematic of memristor-type OS, **b** light-driven conductance switching behaviors, and **c** imaging and pre-processing of UV-light pattern images. **d**–**f** Neuromorphic single-color vison sensor: **d** device schematic of transistor-type OS, **e** light-driven conductance switching behaviors, and **f** imaging and pre-processing of single color pattern images. **g**–**i** Neuromorphic multicolor vison sensor: **g** device schematic of the transistor-type OS, **h** color-driven conductance switching behaviors, and **i** imaging and pre-processing of mixed color patterns. **a**–**c** Reproduced with permission from Ref. [54]. Copyright @ 2019, Springer Nature. **d**–**f** Reproduced with permission from Ref. [55]. Copyright @ 2021, Springer Nature. **g**–**i** Reproduced with permission from Ref. [56]. Copyright @ 2022, Wiley-VCH

Chai et al. [54] were the first to discuss the promising potential of OSs as neuromorphic vision processors for the in-sensor computing of image data. In their study, a neuromorphic vision sensor capable of the in-sensor computing of UV light images was fabricated using a memristor-type OS with a Schottky diode structure of ITO/MoO_*x*_/Pd (Fig. 7a). Upon exposure to UV light, the MoO_*x*_ film underwent a phase transformation into H_*y*_MoO_x_, which is more electrically conductive owing to the light-induced proton ions (H^+^) of the inner water molecules. This led to an excitatory increase in the conductance state from the optical input. Thus, this memristor-type OS could exhibit a UV-light-driven conductance switching function and excitation conductance update behavior under repeated light exposures (Fig. 7b). The original image could be recognized by reading the switched conduction states of the individual devices in the OS array. In addition, by iteratively recognizing the incident image in the neuromorphic vision preprocessor, a well-refined image could be successfully obtained through image sensitivity and noise minimization (Fig. 7c).

Similarly, Sun et al. [55] proposed a neuromorphic vision sensor using a transistor-type OS with a heterogeneous channel (HC) structure (Fig. 7d). The HC transistor-type OS was constructed by vertically stacking carbon nanotubes (CNTs) and a CsPbBr_3_ quantum dot (QD) photoabsorber. The HC structure containing CsPbBr_3_ QD/CNT had functional regions essential for photonic conductance switching behaviors, such as a photoresponsive layer for light absorption (CsPbBr_3_ QD), high-mobility conduction pathway for charge transportation (CNT), and a heterogeneous interface for the nonvolatile storage of photocharges (CsPbBr_3_ QD/CNT). Hence, the conductance state could be updated to different conductance states depending on the light intensity and exposure cycles (Fig. 7e). In addition, when performing neuromorphic recognition of an input image, the transistor-type OS showed a superior photosensitive imaging ability relative to the memristor-type OS, showing the advantage of clear image recognition even with weak and faint image patterns (Fig. 7f). Their work provided an excellent foundation for using OSs as all-in-one neuromorphic vision sensors. However, their OS is difficult to distinguish and recognize various color images due to the limited photosensing spectral range of the CsPbBr_3_ QD photoabsorber (main light wavelength: 516 nm).

To solve this issue, Park et al. [56] reported a color-perceiving OS fabricated using an HC structure, including a multicolor-responsive QD layer (Fig. 7g). Generally, a single-type QD seldom provides clear color sensing selectivity to HC structures. In contrast, a mixed layer of red, green, and blue QDs could introduce an excellent multicolor sensing capability to the HC structure. Here, red CdSe (optical bandgap: 1.86 eV), green CdSe (optical bandgap: 2.15 eV), and blue CdS (optical bandgap: 2.73 eV) QDs covered with Sn_2_S_6_^4−^ ligands were used to make the mixed QD layer. Furthermore, their color-selective photoresponse could be easily manipulated by controlling the mixing ratio. Overall, the mixed QD/IGZO-based OSs exhibit multicolor-responsive conductance switching properties (Fig. 7h), which can be considered as important functions for neuromorphic color vision computing processers. In addition to multicolor recognition, it is also possible to selectively switch the color recognition modes (nonvolatile modes for red–green–blue, green–blue, and blue light detection) by controlling the gate terminal condition. Ultimately, by using an HC transition-type neuromorphic vision sensor with a mixed QD layer, it is possible to selectively recognize and extract a desired image from an input image according to the user's intention (Fig. 7i).

### Transistor-Type Optoelectronic Synapses

Most transistor-type OSs have similar device architectures including insulating gate dielectrics, semiconducting channels, and conductive three-terminal electrodes. However, depending on the device structure, each transistor-type OS plays its own role in performing the conductance switching and analog conductance update functions using optoelectronic programming [57, 58]. To date, three types of transistor-based OSs with unique photoresponsive regions have been designed: (i) HC, (ii) FG, and (iii) non-stoichiometric semiconductor channel (NSC).

#### HC Transistor-Type OS

**Fig. 8:**
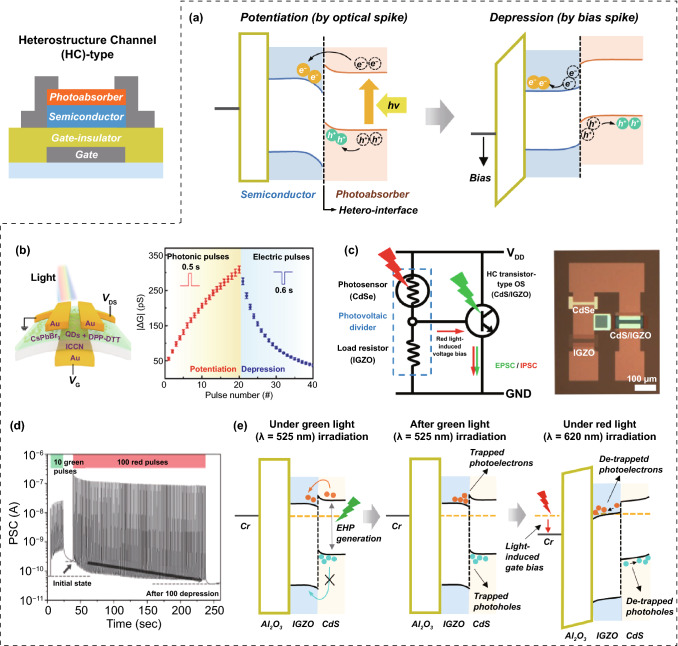
Heterostructure channel (HC) transistor-type OS; **a** operation mechanism, **b** optical potentiation and electrical depression characteristics. Optoelectronic circuit consisting of photovoltaic divider and HC transistor-type OS for all-optical conductance switching function; **c** circuit schematics, **d** green light-induced potentiation and red light-induced depression of channel conductance, and **e** operational mechanism. **b** Reproduced with permission from Ref. [63]. Copyright 2022, Elsevier. **c**, **d** Reproduced with permission from Ref. [66]. Copyright @ 2022, Wiley-VCH

HCs consist of photoabsorbers, high-mobility semiconductors, and hetero-interface regions for light reception, charge transportation, and photocharge storage, respectively (Fig. 8). High-performance photoabsorbers, such as perovskites, organic materials, and metal chalcogenides (which have been highlighted as high-efficiency solar cell absorbers) are preferentially used, owing to their superior sensitivity and low power consumption during vision information processing. Crystalline nanoscale semiconductors or amorphous oxide semiconductors with high mobility are suitable for semiconductor regions to obtain excellent image sensitivity. HC structures can be classified as straddled gaps (type I), zigzag gaps (type II), and broken gaps (type III) [59]. In type I HC structures with Fermi level (*E*_F_) alignment, the conduction band energy edge of smaller bandgap materials (*E*_CS_) is lower than the conduction band energy edge of wider bandgap materials (E_CW_), while the valence band energy edge of smaller bandgap materials (*E*_VS_) is higher than the valence band energy edge of wider bandgap materials (*E*_VW_). That is, *E*_CW_ > *E*_CS_ and *E*_VW_ < *E*_VS_. When light is irradiated, photogenerated electrons and holes in the wider or smaller bandgap materials spontaneously can migrate to the smaller bandgap material side and disappear rapidly through recombination process. Therefore, the type I HC structure is more suitable for light-emitting devices than light-sensing devices. In the case of type III HC structures with E_F_ alignment, *E*_CW_ is lower than *E*_CS_ and *E*_VS_, or *E*_CS_ is lower than *E*_CW_ and *E*_VW_ (‘*E*_CW_ < *E*_CS_ and *E*_VS_’, or ‘*E*_CS_ < *E*_CW_ and *E*_VW_’). However, type III HC structures are rarely formed because the electron affinity between the two materials should be significantly different. In type II HC structures with *E*_F_ alignment, *E*_CW_ is lower (or higher) than *E*_CS_ and *E*_VW_ is lower (or higher) than E_VS_. That is, (i) *E*_CW_ < *E*_CS_ and *E*_VW_ < E_VS_, or (ii) *E*_CW_ > *E*_CS_ and *E*_VW_ > *E*_VS_. Compared with other type HCs, the type II HC is most favorable for the efficient separation of the electron–hole pairs (EHPs) at the hetero-interface and stable storage of the separated photocharges (Fig. 8a). Therefore, many transistor-type OSs are manufactured with type II HCs, including spike-form-bent heterogeneous interfaces [60–63]. When the light signal reaches the photoabsorber, EHPs are formed in the photoabsorber and spontaneously split into photoelectrons and photoholes at the interface between the photoabsorber and the high-performance semiconducting layer due to the presence of type II heterostructures. Since each photocharge is trapped in different layers, the photoelectrons and photoholes can be maintained for a long time without recombination process. Therefore, the channel conductance can be updated into more conductive states by using an optical programming input (Fig. 8b). However, it is difficult to reversibly reduce the channel conductance, even when optical signals with different optical spectra or optical intensities are provided. The channel conductance can only be reversibly reduced using electrical bias signals. Therefore, in general, most OSs utilize both optical and electrical means to perform in-memory computing tasks.

However, several researchers have begun to focus on the development of fully photo-driven OSs in which can change the channel conductance using two kinds of optical signals with different optical spectra [64–66]. Particularly, Park et al. [66] proposed an interesting solution based on the fusion of a photovoltaic divider and an OS for a fully photo-driven transistor-type OS. They manufactured an optoelectronic circuit consisting of a photovoltaic divider (CdSe photosensor and IGZO load transistor) and a CdS/IGZO HC-type OS (Fig. 8c). In fact, the channel conductance of the CdS/IGZO HC-type OS can be updated to more conductive and more resistive values by green light and red light programming signals, respectively (Fig. 8d). When the CdS/IGZO HC-type OS of optoelectronic circuit is exposed to green light, EHPs are generated in the CdS layer (bandgap; 2.36 eV) that can absorb the green light. In contrast, IGZO hardly responds to red and green light due to its wide bandgap (3.69 eV). Here, photoelectrons are spontaneously transferred to the IGZO while photoholes are trapped in the CdS layer due to the presence of a potential barrier. Since the photoelectrons and photoholes can exist separately in different layers, they contribute to the increase of the channel conductance and can be maintained for a long time without recombination even after the green light is removed. Therefore, the CdS/IGZO HC-type OS can exhibit an excitatory update behavior of the channel conductance in the green light programming signal. Next, when red light programming input is applied at the CdSe photosensor, the resistance of the photosensor is reduced due to the generation of photocharges in the CdSe layer (bandgap; 1.7 eV). In contrast, the IGZO load resistor and the CdS/IGZO HC-type OS are insensitive in red light signal because they have a larger energy bandgap than the red light energy. When the resistance of the CdSe photosensor is temporarily reduced by the red light input, the photovoltaic divider passes a positive bias to the voltage output electrode connected to the gate terminal of the HC-type OS (Fig. 8c). The amplitude of the delivered positive bias depends on the resistance ratio between the CdSe photosensor and the IGZO load resistor. In contrast, when there is no external optical signal, almost zero bias is applied to the voltage output electrode because the resistance of the load resistor is set higher than that of the CdS photosensor. Finally, the positive bias transferred from the photovoltaic divider to the gate terminal of the HC-type OS by the red light programming input releases the photocharges accumulated near the heterogeneous interface of CdS/IGZO, reducing the channel conductance of the OS (Fig. 8e).

#### FG Transistor-Type OS

**Fig. 9:**
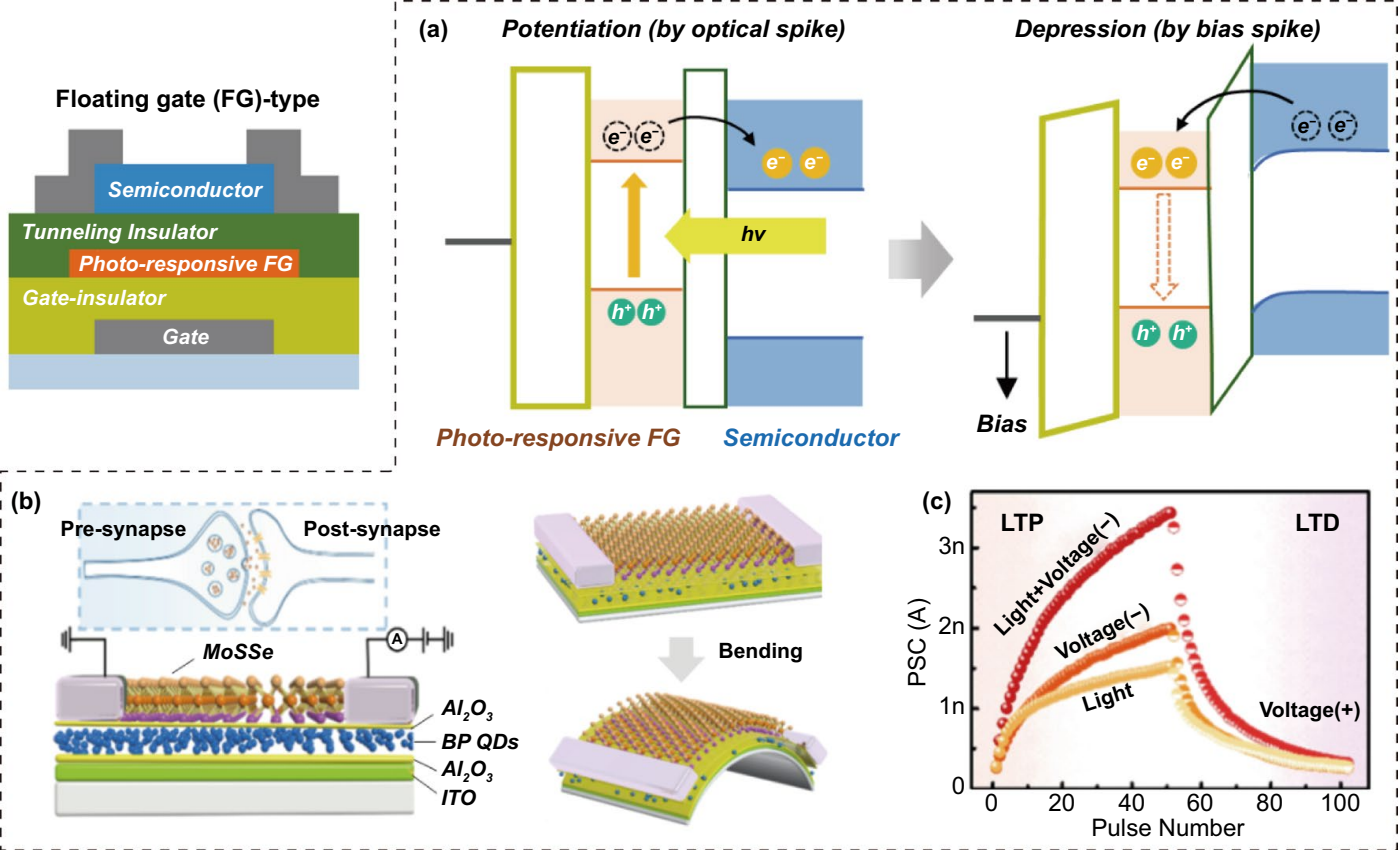
FG transistor-type OS; **a** operational mechanism, **b** black phosphorus (BP) quantum dots (QDs)-FG transistor-type OS, and **c** optical potentiation and electrical depression characteristics. **b**, **c** Reproduced with permission from Ref. [72]. Copyright @ 2021, Elsevier

FG transistor-type OSs can be manufactured by vertically stacking thick blocking dielectrics, photoresponsive semiconducting FGs, ultra-thin tunneling dielectrics, and semiconducting channels with wider bandgap than the FGs (Fig. 9a) [67]. Similar to the other cases, FG transistor-type OSs also utilize optical and electrical means as programming resources for excitatory and inhibitory conductance modulation during in-memory computing. When a light signal with smaller energy than bandgap of semiconducting channel is absorbed at the photoresponsive FG region, EHPs are only formed in FG region through a band-to-band transition. Depending on the band alignment between the FG and channel layer, EHPs can be spontaneously separated or disappeared at FG region. When the conduction band minimum energy level of FG is higher than the that of n-type semiconducting channel, some EHPs can be spontaneously separated due to its band alignment without intentional supply of gate bias. Here, some photoelectrons move across the tunneling layer into the semiconducting channel region and some photoholes are trapped in FG region. The photoelectron supplied through the tunneling layer contribute to the improvement in the conductivity of n-type semiconducting channel region and enable an excitatory conductance update. The excitatory conduction state updated by the optical programming can be maintained for a long time, owing to the photogating phenomenon caused by the photoholes trapped in the FG layer. Meanwhile, the increased channel conductance can be reduced using electrical means. By applying a bias input of the positive polarity to the gate electrode terminal, the charges trapped in the FG region can be removed through a recombination process, thereby enabling a suppressive conduction conversion function.

In FG transistor-type ESs, metallic low-dimensional materials (e.g., Au, Pt, and Cu NPs) are employed in the FG region. In contrast, photoresponsive semiconducting nanomaterials should be employed in the FG region between the insulating blocking and tunneling dielectric layers for the optical conductance updating and nonvolatile switching responses. Various low-dimensional semiconductors with band gaps of visible or near-infrared rays can be utilized in the FG region, such as perovskite QDs, carbon QDs, organic QDs, black phosphorus (BP) QDs, and graphene oxide nanosheets [68–72]. In addition, an ultrathin tunneling dielectric layer with a large bandgap and low defect density is required to prevent recombination between the trapped charge carriers in the FG layer and free carriers in the channel region. In a pioneering survey, Han et al. [71] reported a perovskite QD-based FG OS, including an inorganic CsPbBr_3_ perovskite QD (photoresponsive FG), thick-SiO_2_ (blocking dielectric), thin-PMMA (tunneling dielectric), and p-type pentacene (semiconducting channel). Similarly, Zhang et al. [72] demonstrated a BP QD-based FG OS including BP QDs (photo-active FGs), thick Al_2_O_3_ (blocking dielectric layers), thin-Al_2_O_3_ (tunneling dielectrics), and n-type MoSSe (semiconductor channels) (Fig. 9b). This device was capable of color-driven potentiation and bias-driven depression functions, and demonstrated significant potential for in-memory computing) (Fig. 9c). Regardless of the OS type, an optical signal is usually employed as an excitatory programming input. However, the polarity of the bias signal for depression programming depends on the electrical type of the semiconductor. Generally, a negative (positive) bias signal is suitable for suppressing conductance modulation in an FG transistor-type OS using p-type (n-type) semiconductor channels.

#### NSC Transistor-Type OS

Stoichiometric semiconductors (in which the constituent atoms are bonded in precise integer ratios) exhibit ultrafast optical responses and spontaneous decay characteristics, owing to their direct band-to-band transition and recombination kinetics. Therefore, semiconductor channels with stoichiometric compositions have been preferentially utilized for typical ultra-sensitive transistor-type photosensors without in-memory computing capabilities because of their superior conduction paths and efficient generation kinetics of photocharges [73, 74]. In contrast, NSC semiconductors with a large number of point defects exhibit slow conductance transitions and nonvolatile modulation behaviors under an optical input signal, owing to photo-ionization of the point defects. Therefore, NSC semiconductors are more suitable for the fabrication of transistor-type OSs. There are several ways to obtain NSC semiconductors with many point defects. First, it is possible to artificially introduce point defects into crystalline and amorphous semiconductor materials by incorporating external elements into their interstitial and displacement sites. Second, compared to crystalline materials, amorphous materials inherently contain many photo-ionizable point defect states, owing to their loosely coupled component networks. Therefore, if an amorphous semiconductor with many point defects can be employed in the channel region, an NSC transistor-type OS can be easily manufactured.

**Fig. 10:**
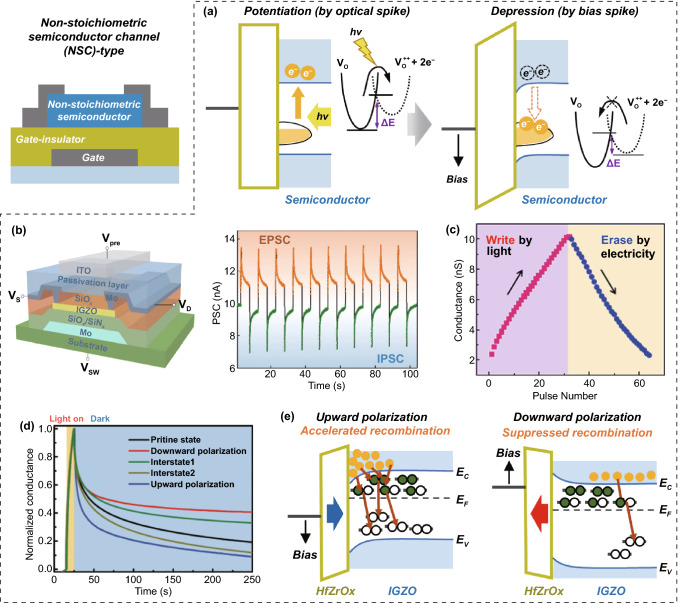
Non-stoichiometric semiconductor channel (NSC) transistor-type OS; **a** operational mechanism. AOS-NSC transistor-type ES using charge trapping structure of SiO_2_/SiN_x_ at gate dielectric; **b** optical potentiation and electrical depression characteristics and **c** linear conductance update behavior. AOS-NSC transistor-type ES using ferroelectric HfZrO_x_ at gate dielectric; **d** retention engineering of optically programmed conductance state and **e** operation mechanism depending on polarization direction of ferroelectric HfZrO_x_. **b**, **c** Reproduced with permission from Ref. [80]. Copyright @ 2019, Royal society of chemistry publishing. **d** Reproduced with permission from Ref. [81]. Copyright @ 2020, Wiley-VCH

As material candidates suitable for the second case, amorphous oxide semiconductors (AOSs) (e.g., InO_*x*_, ZnO_*x*_, SnO_*x*_, IGZO, IZO, and ZnSnO) with n-type electrical properties and excellent conductivity are noteworthy. AOSs inherently have many point defects that cause photo-ionizable subgap states [75, 76]. Among the various point defects, such as *V*_*O*_, metal vacancies, self-interstitial oxygen, and self-interstitial metals, *V*_*O*_ is the most dominantly formed, owing to having the lowest formation energy in n-type AOSs. To date, many researchers have reported a transistor-type OS capable of photo-driven conductance updates and multibit programming operations by using AOSs in the channel region. Under an optical input, the *V*_*O*_-induced deep trap states are broken down into photoelectrons and positively single/double-charged oxygen vacancy ions (Fig. 10a) [77]. The photoelectrons generated from the localized *V*_*O*_ states require high activation energy for non-spontaneous recombination. Through the removal of the optical inputs, these photoelectrons are maintained with slow decay behavior, thereby inducing the excitatory switching of the channel conductance. Meanwhile, electrical programming is used for the inhibitory conductance modulation. When an electrical bias of appropriate polarity is applied to the gate terminal region, it can activate the recombination process between the photocharge and ionized V_O_ states, converting the channel conductance to a more resistive state. In particular, a positive-bias signal for inhibitory modulation is suitable because AOSs have n-type semiconducting characteristics. Generally, in gate dielectric region of transistors, typical single material-based high-quality insulators such as SiO_2_ and Al_2_O_3_ are mainly employed. However, in these cases, high voltage and long-term programming conditions are mainly required to recombine the photocharge and the ionized *V*_*O*_ state. [78, 79]. It makes difficult to realize OSs with linear conductance switching and power-efficient computing performance. To address this issue, it can be good approach to introduce additional charge trapping sites at the channel/gate dielectric interface or gate dielectric bulk region of the AOS-based OSs. In particular, during electrical bias-induced depression programming, photocharges can be efficiently removed through a more frequent recombination process at additional charge trapping sites. Indeed, Miao et al. reported an AOS-based OS with a charge trapping structure of SiO_2_/SiN_*x*_ in the gate-dielectric region (Fig. 10b). Despite the adoption of a weak electrical programming signal, photocharges generated in the channel region can be easily removed by efficient recombination action in the charge trapping region, allowing linear and symmetric conductance updates (Fig. 10c) [80].

Next, in general, AOS-based OSs exhibit unstable retention of optically programmed channel conductance with gradual attenuation behavior (Fig. 10d, pristine state). However, Lee et al. [81] devised a ferroelectric-assisted AOS-based OS which introduced ferroelectric HfZrO_*x*_ layer in the gate dielectric region for long-term nonvolatile storage characteristics of optically-programmed channel conductance. Here, the long-time retention behavior of the optically programmed channel conductance state could be controlled depending on the polarization direction of the ferroelectric layer (Fig. 10d). Compared to the case of the upward polarization formed by applying the temporary positive gate bias pulse (+ 5 V, 10 ms), the optically programmed channel conductance could be robustly maintained when the ferroelectric HfZrO_*x*_ layer had a downward polarization state formed by the temporary negative gate bias pulse (− 5 V, 10 ms). This is because the presence of downward polarization of HfZrO_*x*_ can dramatically suppress the recombination reaction between photoelectrons and ionized oxygen vacancies in the IGZO channel (Fig. 10e). Although nonvolatile operation of excitatory conductance state formed by optical programming could be successfully realized in the ferroelectric-assisted AOS-based OS, there is no consideration about depressive programming of conductance state by electrical bias. Thus, the research to address this issue is essential. The dual-gate transistor architecture will become one of good approach. A ferroelectric material should be utilized in one gate dielectric region for nonvolatile engineering of an optically programmed conduction state and a charge trapping structure should be utilized in the opposite gate dielectric region for inhibitory conduction update via electrical bias programming.

### Memristor-Type Optoelectronic Synapses

Regarding memristor-type OSs, Schottky barrier (SB)- and photogating memristor-type OSs have been widely developed. They have a simple two-terminal device structure with a photoresponsive active space between a transparent conductive TE and metallic BE.

#### SB Memristor-Type OS

**Fig. 11:**
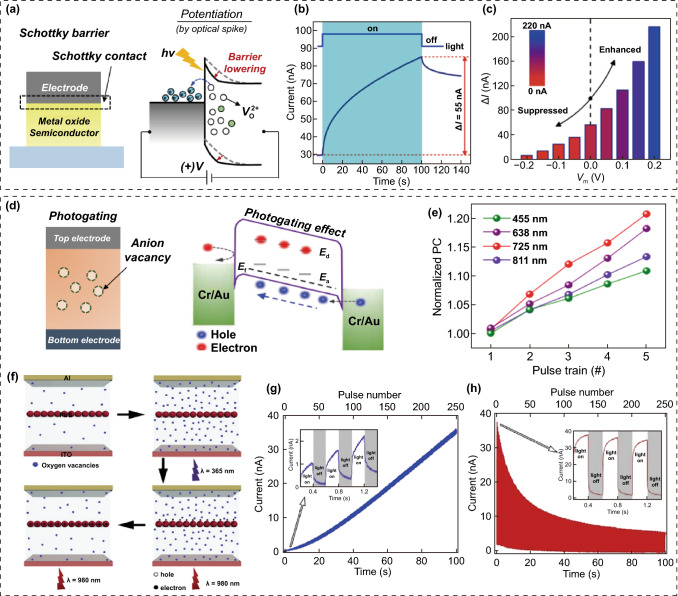
Memristor-type OSs; device configuration, operation mechanism and optoelectronic conductance switching performance. Schottky-type memristor-type OS; **a** operation mechanism, **b** optical potentiation, and **c** conductance switching performance engineering using bias modulation. Photogating memristor-type OS with antimony vacancies; **d** operation mechanism and **e** optical potentiation as a function of light wavelength. Fully optically modulated photogating memristor type OS with oxygen vacancies and QDs; **f** operation mechanism, **g** UV light-driven potentiation, and **h** IR light-driven potentiation. **b**, **c** Reproduced with permission from Ref. [86]. Copyright @ 2019, American Chemical Society. **d**, **e** Reproduced with permission from Ref. [87]. Copyright @ 2021, AAAS. **f**–**h** Reproduced with permission from Ref. [91]. Copyright @ 2019, Elsevier

SB memristor-type OSs have a Schottky contact between a semiconductor active layer (usually n-type semiconducting metal oxides, e.g., ZnO, CeO, and SnO) and conductive TE (usually transparent conductive oxides (TCOs), for example, ITO and IZO) and an ohmic contact between the active layer and bottom metal electrode (Fig. 11a) [82–86]. In the initial state, an SB memristor-type OS using a semiconductor oxide material for the active layer region exhibits a low conduction state owing to the high SB and wide depletion region formed in the Schottky junction. When the device is exposed to an optical spike, the photoelectrons and charged oxygen vacancies generated in the semiconducting oxide layer via the photoionization process can contribute to the generation of an excitatory conductance update (Fig. 11b). This is because the photoionized *V*_*O*_s reduce the barrier height and depletion width at the Schottky junction. Li et al. [86] demonstrated an artificial OS based on a Schottky junction structure using ITO (a TCO) and Nb-doped SrTiO_3_ (an n-type metal oxide semiconductor). The device could exhibit excitatory conductance-switching dynamics (i.e., potentiation) under optical programming spikes. In addition, by controlling the modulation bias conditions, the photo-ionized *V*_*O*_s generated from the light stimulus and height of the potential barrier could be effectively modulated to control the conductor switching performance. Irradiating the device with light while applying a positive modulation bias to the ITO TE could allow for photoionizing more oxygen vacancies, leading to a stronger conductivity transition while lowering the barrier height of the Schottky junction (Fig. 11c).

#### Photogating Memristor-Type Oss

Photogating memristor-type OSs usually have space charge regions such as localized subgap sites and/or heterogeneous spaces within the active layer [87–89]. The carriers trapped in the space charge region can induce an electric field, which serves as a photogate terminal. In general, localized subgap states can be caused by point defects [87, 88], and heterogeneous spaces can be formed in the heterogeneous compositional phases and multilayered heterostructures [89, 90]. Yang et al. [87] reported photogating memristor-type OSs using p-type semiconducting SnS, including cation and anion vacancies in the active region (Fig. 11d). When the device was exposed to optical programming spikes, optical carriers were created in the active layer. Some photoelectrons were trapped in the local donor state induced by the sulfur vacancies, creating a negative photogating effect. More hole carriers were induced in active layer by the photogating effect, increasing the conductivity of p-type semiconductor-based memristor. In addition, the device exhibited color-programmable in-memory computing behaviors, owing to the small bandgap of the SnS (Fig. 11e). Han et al. [91] reported a photogating memristor-type OS fabricated using PbS QDs and a n-type ZnO film. The ZnO film contained numerous oxygen vacancies, and there were many heterojunctions between the ZnO film and PbS QDs (Fig. 11f). A device with a heterojunction structure with PbS QDs interposed between ZnO active layers allows for excitatory (by ionization of the *V*_*O*_) and inhibitory (by neutralization of the ionized *V*_*O*_) switching in conductivity via UV or near-infrared programming spikes, respectively. Under UV illumination (*λ* = 365 nm), the *V*_*O*_ of the ZnO layer is photoionized and acts as a locally positively charged photogate site, leading to a gradual increase in conductivity (Fig. 11g). In contrast, under infrared illumination (*λ* = 980 nm), the photoelectrons generated from the PbS QDs migrate to the ZnO layer to neutralize the ionized *V*_*O*_s and suppress the conductivity modulation (Fig. 11h).

## Advanced Applications of Neuromorphic Vision Sensors

Recently, researchers are seeking further advances beyond the current functional and imaging levels of neuromorphic vision sensors for machine vision. For image recognition, researchers are developing neuromorphic vision sensors with advanced features, including adaptive recognition, focus recognition, and selective focus reception, as derived from superior biological visual recognition systems. Just as living things use visual information as a resource for decision-making, researchers are working to develop neuromorphic vision sensors for decision-making, such as for collision avoidance, nociceptive movement, and nociceptive protection.

### Environment-Adaptable Neuromorphic Vision Sensors

**Fig. 12:**
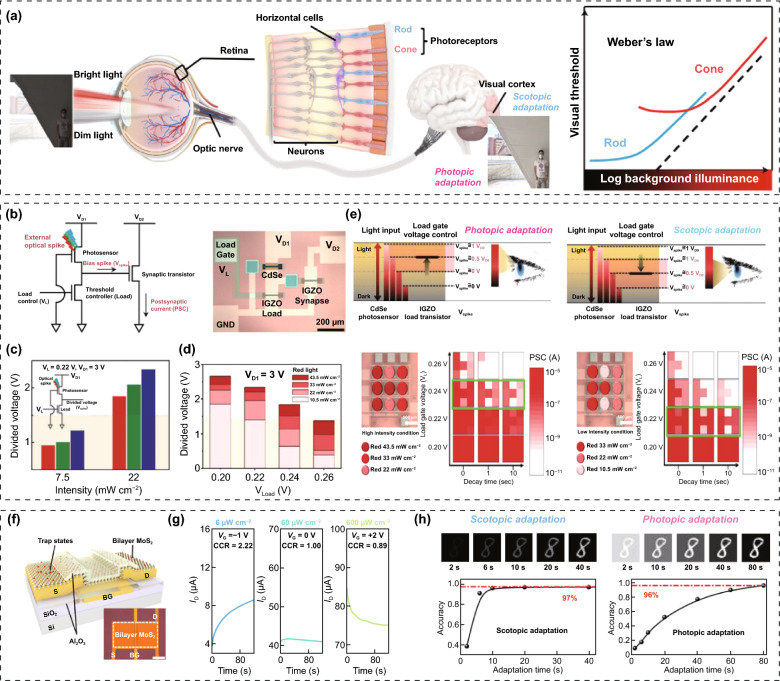
Environment-adaptable neuromorphic vision sensors. **a** Environment-adaptable biological vision system consisting of rod and cone cells (Weber’s law, photopic and scotopic adaptation). **b**–**e** OSC-type environment-adaptable neuromorphic vision sensor: **b** circuit schematic, **c** light-to-bias conversion performance of photovoltaic divider, **d** the change of light-adaptation mode through load gate voltage control, and **e** the image perception on photopic and scotopic adaptation modes. **f**–**h** OS-type environment-adaptable neuromorphic vision sensor: **f** device schematic, **g** the change of light-adaptation mode through load gate voltage control, and **h** image perception on photopic and scotopic adaptation modes. **a**, **f**–**h** Reproduced with permission from Ref. [95]. Copyright @ 2022, Springer Nature. **b**–**e** Reproduced with permission from Ref. [96]. Copyright @ 2019, Wiley-VCH

The human retina, as a biological image detector, consists of biological cells such as photoreceptors (rods and cones) and horizontal cells (Fig. 12a) [92, 93]. The cone and rod cells are responsible for color perception and the contrast sensing of incident light, respectively. These biological photoreceptors have a low dynamic range for light detection compared with current electronic image detectors; such a range is disadvantageous for high-resolution image recognition. However, in practice, the human retina can clearly recognize objects in a much wider range of light (from much darker to much brighter) than electronic image sensors by using visual adaptation functions [94]. There are two cases of biological visual adaptation for accurate image recognition in different ambient-light environments: photopic adaptation and scotopic adaptation. When changing from a bright place to a dark environment, the retina can initially see very few objects. However, dark objects can be gradually recognized through a scotopic adaptation process that lowers the visual threshold and activates photosensitive rod cells instead of cone cells. In contrast, our retinas are initially dazzled by bright objects when exposed to bright light. However, it is possible to gradually recognize bright objects by suppressing ambient light noise through a photopic adaptation process that activates photosensitive cone cells and enhances the visual threshold. Mimicking biological visual adaptation, in which the retina's sensitivity is automatically adjusted to the ambient light environment, is currently a key element for building advanced image-sensing systems with wider detection ranges and more accurate recognition using electronic image sensors.

Recently, many efforts have been made to build neuromorphic image sensors able to adapt to ambient-light environments [95–103]. In a pioneer study on circuit-type neuromorphic image sensors, Park et al. [96] reported an OSC constructed by the interconnection of a photovoltaic divider for photo-sensing and a transistor-type ES for in-memory computing (Fig. 12b). The transistor-type ES was fabricated using an inorganic solid electrolyte (Na-Al_2_O_3_) and semiconducting channel (IGZO). Meanwhile, the photovoltaic divider, comprising a photosensor and load transistor, was responsible for the visible-light reception and photo-to-bias conversion. The optical signal was converted into a bias signal in the photovoltaic divider, and the amplitude of the converted bias signal was proportional to the intensity of the incident light. And then, the converted bias signal was transmitted to the gate terminal of the transistor-type ES (Fig. 12c). The bias spike induced a nonvolatile modulation of the channel conductivity, owing to the electrochemical doping of the semiconductor channel by mobile Na^+^ ions. In general, for excessively bright images with a large amount of ambient noise, it is difficult to detect an accurate image, because the channel conductance of every transistor-type ES in every pixel is updated indiscriminately. In contrast, for very faint images with low intensity, the pattern images are difficult to detect because the channel conductance of all transistor-type ESs in all pixels rarely exceed the threshold (Fig. 12d). However, switching the visual adaptation mode by controlling the load-gate bias can result in an overall increase or suppression of the amplitude of the transformed bias spike under the same optical stimulus (Fig. 12e). In a bright-light environment, image recognition can be better achieved by increasing the load gate bias (photopic adaptation mode), whereas faint images in a dark–light environment can be distinguishable by decreasing the load gate bias (scotopic adaptation mode). Another study reported OSCs with similar architectures consisting of MAPbI_3_ perovskite photosensors, IZO load transistors, and polymeric electrolyte transistor-type ESs [97]. One notable advance is that more accurate image pre-processing and spontaneous recovery can be achieved in neuromorphic image sensors by using habitual visual processing implemented by introducing a pulse-like load bias instead of the conventional constant load bias condition. A circuit-type neuromorphic image sensor can achieve high-performance image recognition over a wide dynamic range through its adaptive function. However, challenging problems remain, e.g., concerning the multiple components, complex integration processes, and low pixel density.

To overcome these challenging issues, researchers have developed OS-based neuromorphic vision sensors able to improve the image recognition performance by introducing an adaptive function. In general, for the manufacture of high-performance phototransistors, it is ideal to introduce a high-crystal semiconductor and minimize the charge trap states in the channel region. During photosensing, the charge-trapping sites in the photosensor mainly play negative roles, such as slowing down detection and reducing sensitivity. However, some researchers have intentionally introduced charge trap sites within the device, which enables the development of environmentally adaptive photosensors based on sub-linear photoresponse properties [104]. Here, sub-linear photoresponse means that the magnitude of the photocurrent generated from the low (high) light input is enhanced (reduced), compared to the case where the photocurrent and the incident light intensity have a linear relationship. Chai et al. [95] developed MoS_2_ phototransistors with an environment-adaptive image-sensing capability (Fig. 12f). Many charge trap sites in the channel/insulator interface region were intentionally introduced to enable the photopic and scotopic sensing operations. The visual adaptation mode could be activated by applying a constant gate bias to the gate terminal, and the adaptive performance can be transformed by changing the gate bias value (Fig. 12g). When a positive gate bias was applied, the photosensor became insensitive (photopic adaptation mode). Here, the photocurrent level of MoS_2_ phototransistors with sub-linear photoresponse characteristic can be gradually decreased with repeated exposure to a strong light input. This is because some of the photocharges generated in the channel are continuously trapped at the charge trap sites in the channel/insulator interface region by repeated strong light irradiation, contributing to the gate bias shielding. Therefore, it became more suitable for detecting bright images with high noisy background. In contrast, when a negative gate bias was applied to the gate terminal, the photosensor became more sensitive (scotopic adaptation mode). The photocurrent level can be progressively enhanced with repeated exposure to a weak light input. This is because photocharges can be stably generated and transferred to the electrode terminals without gate shielding by suppressing charges trapped in the interface region using a negative gate bias. Thus, it became more suitable for detecting dark images in dim light environments (Fig. 12h). Another study reported a HC transistor-type OS with a photopic adaptation function constructed using CsPbBr_3_ QDs and a MoS_2_ semiconductor channel [98]. The defective heterogeneous interface regions provided a mechanistic resource for charge trapping and de-trapping for adaptive sensing functions. When an optical signal arrived at the MoS_2_ channel region, the photocarriers initially contributed to the excitatory conductance update; however, some experienced charge trapping at the heterogeneous interface. Therefore, the excitatory conduction state gradually returned to its original state, and this behavior was accelerated in the photopic adaptation mode with a positive gate bias applied.

By incorporating environmental adaptation capabilities into neuromorphic image sensors, the potential for high-speed and low-power pre-processing for image recognition can be dramatically improved. However, the major limitation of currently developed adaptive neuromorphic sensors is that they cannot automatically switch their adaptive mode according to the ambient lighting environment. Therefore, new hardware or software methodologies for the automatic conversion of adaptive modes must be developed for advanced artificial machine vision systems.

### Neuromorphic Collision Sensor

**Fig. 13:**
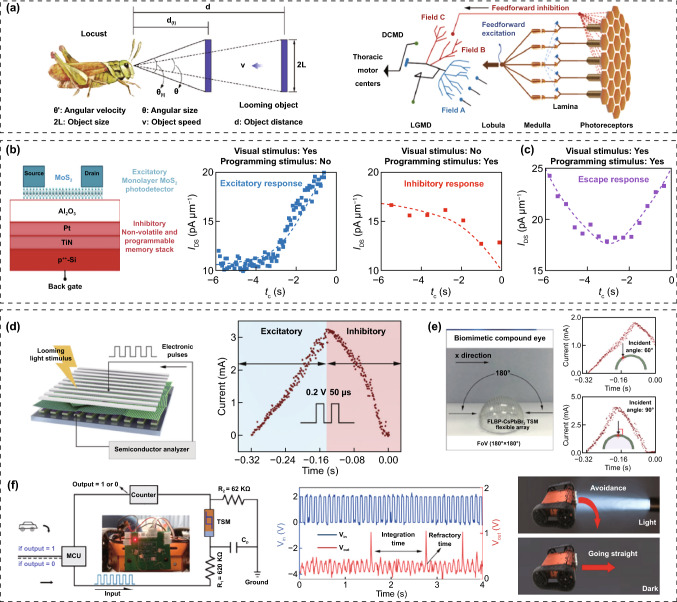
**a** Biological collision avoidance system. **b**, **c** Neuromorphic collision sensor using MoS_2_ photodetector and FG transistor-type ES: **b** device schematics with light-driven exhibitory and bias-driven inhibitory functions for collision sensing and **c** characterization curve for collision-avoidance decision-making. **d**–**f** Neuromorphic collision sensor using memristor-type OS: **d** device schematics, **e** position-dependent collision detection performance of collision sensors placed on compound eye, and **f** proof-of-concept of a collision avoidance system built using a neuromorphic collision sensor and an Arduino microcontroller. **b**, **c** Reproduced with permission from Ref. [107]. Copyright @ 2020, Springer Nature. **a**, **d**–**f** Reproduced with permission from Ref. [108]. Copyright @ 2021, Springer Nature

As a biological proximity detector, the bio-visual system can recognize distances from objects by measuring changes in the frequency and intensity of the light signal in the visual information reflected from the objects. Based on the proximity detection function, the human vision system can perform advanced imaging and decision-making tasks such as collision avoidance from approaching dynamic objects and stereoscopic and focal image detection of static objects. For collision avoidance, biological proximity recognition systems employ several strategies for estimating the approximate distance from an approaching object. In the case of a human vision system, the human eye perceives distances from objects using the binocular parallax principle [105, 106]. As the eyes of humans are far apart from each other, the image coming into each eye will look slightly different. By comparing and analyzing this two-image information, the brain can accurately recognize the shape, movement, and distance of an object. Insects have compound eyes made up of several lenses, which allow for wide field-of-view detection. However, insects cannot use the human distance recognition method with binocular parallax, because the distance between their eyes is too short. Instead, the distance is estimated based on the speed at which the object approaches the compound eye (Fig. 13a). Collision-monitoring neurons in the insect visual system have an excitatory synaptic portion for responding to the angular velocity of an approaching object, and an inhibitory synaptic portion for responding to the angular size of the approaching object [106, 107]. As an object approaches, the angular velocity and size of the approaching object gradually increase. An increase in angular velocity introduces a gradual excitatory modulation in the collision-monitoring neuron. In contrast, an increase in the angular magnitude introduces a gradual inhibitory modulation. Owing to the opposing update behaviors occurring continuously as the distance to the approaching object gets closer, the accumulated weight value or firing frequency in the neuron reaches its maximum peak at any critical moment just before the collision. When this maximum peak point appears, the decision-making for collision avoidance should be performed.

Das et al. [107] reported neuromorphic collision sensors for mimicking proximity recognition, and an automated decision-making method for collision avoidance in insect vision systems. The neuromorphic collision sensors were capable of optical excitation and electrical suppression updates of the channel conduction states, owing to the use of MoS_2_ semiconductor channels and the FG device structure (Fig. 13b). In this approach, the intensity of light reflected from the object increased as the object approached, resulting in the monotonically increasing conductivity of the MoS_2_ semiconductor with an excitatory optical feed-forward stimulus. Meanwhile, as the angular size increased as the object approached, the suppressive electrical feed-forward stimulus applied to the gate terminal of the neuromorphic collision sensors induced a continuous monotonic decrease in the channel conductance. Thus, as the object approached, the visual excitation and electrical suppression of the neuromorphic collision sensor competed, resulting in a nonmonotonic conductance update. By monitoring the appearance of the maximum peak in the conductance of the neuromorphic collision sensor, it was possible to determine when a collision was nearly imminent (Fig. 13c). Thus, when the minimum peak was observed, it was necessary to judge the next appropriate action and take a collision avoidance action accordingly. Han et al. [108] demonstrated an autonomous collision avoidance system using a neuromorphic collision sensor in the form of a memristor-type OS and spiking neuron circuit. The neuromorphic collision sensor with the memristor-type OS configuration consisted of an Ag TE, a few-layer BP-CsPbBr_3_ perovskite QD (FLBP-CsPbBr_3_) heterogeneous material, and an ITO BE (Fig. 13d). This memristor-type OS was capable of an excitatory response to the feed-forward stimulus of an electrical bias spike and an inhibitory response to the feed-forward stimulus of the light reflected from an approaching object. It was confirmed that a maximum current peak could be generated in the neuromorphic collision sensor at the critical moment of collision. This is because metallic CFs were formed from electrically biased programming spikes and then gradually dissolved by stronger light stimulation of an approaching object. In particular, omnidirectional collision detection was achieved by integrating a collision sensor array onto a circular polydimethylsiloxane lens (Fig. 13e). Finally, a robotic car with automatic decision-making for collision avoidance was successfully demonstrated using the memristor-type collision sensor and spiking neuron circuit (Fig. 13f).

Although the development of neuromorphic vision sensors for collision avoidance is exciting, it is still in its infancy. In collision avoidance, the processing speed for the optical information from an object is a more important factor than the recognition accuracy of the shape. Therefore, it is necessary to steadily devise architectures and material selections to accelerate the data-processing speeds of neuromorphic vision sensors.

### Neuromorphic Nociceptive Sensor

**Fig. 14:**
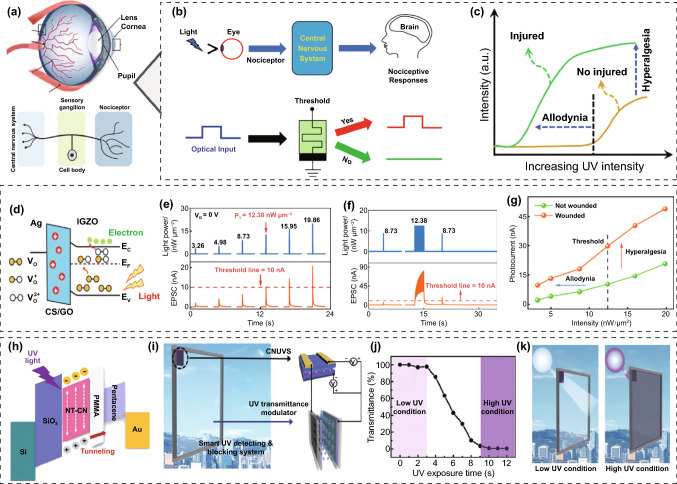
Neuromorphic Nociceptive Sensor. **a** biological and **b** biomimetic nociceptive sensing system and **c** schematic characterization of nociceptive allodynia and hyperalgesia behaviors. **d**–**g** Metal oxide-based photonic nociceptor: **d** energy band structure and working mechanism, **e** light intensity-dependent threshold behavior, **f** threshold behavior depending on combination of the stimulus condition (intensity and pulse number), and **g** the allodynia and hyperalgesia characteristics. **h**–**k** Smart system using a neuromorphic photosensor and a transmittance modulator for UV protection: **h** energy band structure and working mechanism, **i** UV-light-blocking smart window system **j** UV transmittance of the system as a function of UV exposure time, and **k** schematic proof-of-concept of the system. **a** Reproduced with permission from Ref. [109]. Copyright @ 2020, Springer Nature. **b**, **c** Reproduced with permission from Ref. [118]. Copyright @ 2019, Wiley-VCH. **d**–**g** Reproduced with permission from Ref. [119]. Copyright @ 2021, Wiley-VCH. **h**–**k** Reproduced with permission from Ref. [120]. Copyright @ 2020, Wiley-VCH

To protect human skin and organs (especially the eyes) in daily life, it is necessary to detect harmful signals such as UV rays and radiation. However, it is difficult to immediately recognize latent damage, as UV rays are invisible to the human eye. Several studies have recently reported artificial neuromorphic nociceptor sensors for mimicking pain-sensory biological nociceptors that detect and transmit noxious stimuli to the central nervous system (Fig. 14a-b) [109–117]. In the process of pain perception, an external stimulus input above a threshold can be perceived; this is called the threshold behavior. After stimulation, nociceptors gradually return to their initial state over time. This is called the relaxation behavior. During the relaxation process, if an additional stimulus is applied before the residual response completely disappears, the nociceptor may be more sensitive to external stimuli, and may even respond to subthreshold stimuli. Such sensitization can be extended to allodynia and hyperalgesia (Fig. 14c). Under high-intensity stimulation, nociceptors can be easily injured, in which state they respond strongly to mild stimuli. Specifically, in the damaged state of the nociceptors, an allodynia behavior that responds to a stimulus intensity below a threshold and hyperalgesia behavior that responds to a stimulus more strongly may occur.

Wide-bandgap material-based photonic nociceptive devices have been studied with the goal of selectively perceiving harmful incident UV rays in artificial vision systems. Kim et al. [118] demonstrated a metal oxide memristor-type artificial photonic nociceptor in which an Sb-doped SnO_2_ active layer was employed. In addition to perceiving deleterious UV light, the device emulated a variety of nociceptive functions, including allodynia and hyperalgesia. The device structure consisted entirely of metal oxide materials (ZnO, Sb-doped SnO_2_, and F-doped SnO_2_) for transparent optoelectronic applications. In general, the harmful light perception function is expected to be utilized as a practical alert system for providing notifications regarding the accumulation of potential light damage. Recently, an NSC transistor-type OS-based photoreceptor for artificial vision with an alerting function for harmful light was reported [119]. The channel conductance of the IGZO NSC transistor-type OS could be increased because of carrier generation owing to band-to-band excitation and photoionization of the V_O_ subgap state. It is known that the photoionization of V_O_ generally triggers the phenomenon of persistent photoconductivity, which in turn triggers the photosynaptic operation of the device (Fig. 14d). Therefore, the channel conductance of the nociceptors could be increased with increasing light-stimulus intensity (Fig. 14e). In addition, the nociceptor device could be damaged (injured) by stimulation accumulation (increasing the number of stimulation pulses), after which pain perception (threshold behavior) was possible under sub-threshold stimulus intensities, as well as increased response levels under above-threshold intensity stimuli (Fig. 14f). Thus, allodynia and hyperalgesia behaviors were observed (Fig. 14g). It was also confirmed that the pain perception could be modulated by changing the gate bias conditions to control the number of carriers accumulated at the interface between the channel and gate dielectric. Photoresponsive neuromorphic nociceptor sensors can enable intelligent automatic protection systems against harmful light signals.

In a pioneering study, Lee et al. [120] proposed a UV-light-blocking window system using UV-light-responsive neuromorphic nociceptors and UV transmittance modulators. In this study, an FG transistor-type OS was fabricated by vertically stacking nitrate-treated C_3_N_4_ (*E*g = 3.41 eV), PMMA, and a pentacene semiconductor for a UV-light-responsive artificial photon nociceptor (Fig. 14h). Under UV stimulation (*λ* = 365 nm), the C_3_N_4_ layer acted as a negative photogate by trapping photogenerated electrons, thereby increasing the channel conductivity of p-type pentacene. In contrast, the UV transmittance of the modulator was altered according to the supplied variable bias arising from the UV-light-responsive artificial photon nociceptors (Fig. 14i). Thus, the proof-of-concept showed that a neuromorphic nociceptive system integrated with a UV transmittance modifier could monitor harmful UV rays and automatically block UV rays when a threshold was exceeded (Fig. 14j–k). Integrating harmful light monitoring and protection into artificial vision systems provides intelligent features for preventing skin and eye aging and related diseases.

In order to put the nociceptive function of neuromorphic nociceptive sensors into practical use, the key factors for automatic decision-making should be considered such as strong and timing of nociceptive response, power consumption, and selective response about intensity and spectrum of optical and electrical stimuli. In particular, the threshold setting of nociceptive sensors, which is the criterion for determining the initiation of nociceptive responses, should be designed according to the onset conditions of the following actuating or protective devices. For effective nociceptive behavior in real life, it may be necessary to set the threshold value quantitatively and precisely by associating cumulative levels of incoming external stimuli that begin to substantially affect humans or other receivers in various surrounding environments. In addition, system integration of nociceptive sensors and output devices such as actuators, smart glasses and light-emitting devices is required to implement the ability to actively avoid or protect against harmful stimuli through automatic recognition and decision-making. Several artificial nociceptor-based smart systems have been proposed, but it still remains a major challenge.

## Outlook

In this review, we summarized the recent progress and advanced functional applications in neuromorphic vision sensors. The representative characteristics of neuromorphic vision sensors based on near-sensor and in-sensor computing was summarized in Table 1. Although significant progress has been made in OSC- and OS-based neuromorphic vision sensors, several major issues and challenges remain, and require further study.

**Table 1:** Characteristic summary of neuromorphic vision sensors for near-sensor and in-sensor computing

Parameters	Near-sensor computing	In-sensor computing
Hardware implement	Optoelectronic synaptic circuit	Optoelectronic synapse
Essential hardware parts	Photosensor, electrical synapse, photovoltaic transducers	Optoelectronic synapse
Signal processing sequence	Light input → bias (conversion) → conductance switching → current readout	Light input → conductance switching → current readout
Device density	Low (many parts)	High (All-in-one)
Power consumption	High	Low
Programming speed	Low (signal transmission between parts)	High (Direct conductance update)

In the case of OSCs, the planar and vertical serial mode frames of photosensors and in-memory computing processors are widely used to indirectly modulate the conductance of adjacent ESs after capturing visual information from photosensors. Therefore, intrinsic problems exist, such as excessive numbers of parts, complex manufacturing processes, and low device density. To realize higher resolution and smaller chip size, compact design of individual components and overall circuits and further development of precision manufacturing techniques are required. In addition, when light spikes are repeatedly incident on the photosensor in the OSC, some amplitude of the voltage output signal supplied from the photovoltaic divider to the voltage output electrode may be lost due to the problem of impedance mismatch between elements [121, 122]. This problem becomes more serious in the high-frequency optical spikes condition. Here, the information loss and noise increase in OSCs by impedance mismatching will cause distorted imaging task and low image recognition accuracy. Thus, in the future, the impedance matching design between the functional parts in OSC-type neuromorphic vision sensors is essential considered for high-frequency image information processing.

OSs are advantageous for high-resolution and fast image pre-processing because of their high-density fabrication, shorter data processing paths, and fewer components relative to OSCs. However, because the OS is an all-in-one device, it is difficult to simultaneously optimize the light reactivity, spectral selectivity, conductivity duration, and power consumption efficiency. Therefore, many studies, including those on material discovery, device architecture design, and self-power operation, must be continuously performed for neuromorphic image pre-processing tasks, including contrast enhancement, noise suppression, fast recognition, and spectral selectivity. Nanomaterials have mainly been used, but they are disadvantageous for device stability and uniform array production. In addition, is difficult to develop a high-resolution neuromorphic vision sensor using a transistor-type case (as opposed to a memristor-type case). Thus, it is necessary to develop high-density manufacturing technologies (e.g., e-beam and nanoimplant lithography) and fine device structure designs (e.g., nanogap, vertical, or three-dimensional channel structures).

To date, many neuromorphic vision sensors have been built on rigid substrates and have generally focused only on demonstration and performance optimization for neuromorphic processing of visual information. However, to implement eye-shaped curved vision systems, wearable or skin patched or implantable optoelectronic systems, and soft robots, neuromorphic vision sensors must be fabricated on flexible or stretchable substrates for freeform platforms. Therefore, many studies should be performed for the realization of freeform neuromorphic vision sensors: (i) low-temperature processing (e.g., e-beam treatment, deep UV light treatment and microwave treatment), (ii) deformable material adaptation (e.g., low-dimensional; materials, organic materials, and liquid metals), and (iii) mechanically robust structural architectures (e.g., nanostructuring, rigid island, and Kirigami structure).

In the future, it will be necessary to develop a neuromorphic vision sensor able to recognize 3D image information beyond current 2D plane image recognition. In addition, there is a need to accelerate the development of intelligent robots and autonomous driving mobility by building an intelligent vision system with active decision-making and passive protection functions based on neuromorphic vision sensors beyond image recognition.
